# A251 NON-ALCOHOLIC FATTY LIVER DISEASE AT A CANADIAN TERTIARY CARE CENTRE: RISK FACTORS AND SEVERITY OF DISEASE

**DOI:** 10.1093/jcag/gwac036.251

**Published:** 2023-03-07

**Authors:** N K Klemm, K Zhu, A Jaffer, A Ramji, A Mohajerani

**Affiliations:** 1 Gastroenterology; 2 Medicine, University of British Columbia; 3 Gastrointestinal Research Institute; 4 University of British Columbia, Vancouver, Canada

## Abstract

**Background:**

Non-alcoholic fatty liver disease (NAFLD) is the leading cause of liver disease worldwide with an increasing prevalence of 25-40%. Although the prevalence increases to 57.5-74% in obese patients, lean individuals also develop NAFLD. Patients are commonly asymptomatic and the diagnosis is often incidental or when progressed to cirrhosis. Once fibrosis has developed, the risk of cardiovascular and liver-related death increases exponentially. The increasing prevalence of NAFLD presents significant healthcare and economic consequences.

The severity of NAFLD and its risk factors have been studied in various countries, which guide decisions on screening and management. Similar studies have not been performed in Canada.

**Purpose:** To determine the severity of NAFLD in a tertiary care centre and associated risk factors.

**Method:**

Retrospective review of patients with NAFLD diagnosed on ultrasound, or fibroscan from January 1, 2019 to December 31, 2021. Patients were 18 years or older and were excluded if they had co-existent liver disease, significant alcohol use or CAP <238. CAP and fibrosis scores were determined using transient elastography. Chi-square and multivariate analysis were performed for statistical analysis.

**Result(s):** A total of 583 patients were included in the study; 312 (53.5%) were male and the mean age was 54.04 years. The majority of cases, 317 (56.8%), were diagnosed by ultrasound or CT scan and only 30 (5%) patients had a known family history of NAFLD. Lean-NAFLD (L-NAFLD) was present in 83 (15.2%) patients, overweight NAFLD (OW-NAFLD) 220 (40.2%), obese-NAFLD (OB-NAFLD) 206 (37.7%) and morbidly obese-NAFLD (MB-NAFLD) 38 (6.9%).

The prevalence of T2DM was 28.6%, dyslipidemia 37.4%, hypertension 35.5%, coronary artery disease 6.2% and obstructive sleep apnea 6.7%. Risk factors for Stage 3 steatosis (CAP>290) included BMI>30 (2.84) and type 2 diabetes (OR 2.45), but not dyslipidemia, hypertension, age or gender. Type 2 diabetes, dyslipidemia, hypertension, BMI, gender and age were not significantly predictive of moderate steatosis (CAP 260-289).

The proportion of patients with transient elastography scores of F2, F3, and F4 were 16.0%, 7.7%, and 11.0%, respectively. F2 and F3 scores were associated with T2DM (OR 1.94), BMI > 30 (OR 1.83, p<0.05) and age >60 (OR 1.90, p<0.05), but not dyslipidemia or hypertension. F4 scores were significantly associated with age > 60 (OR 1.89), T2DM (OR 2.60), dyslipidemia (OR 1.79), hypertension (OR 1.86) and BMI>30 (OR 2.54).

**Conclusion(s):**

Diabetes and BMI were associated with severe steatosis and fibrosis. Dyslipidemia and hypertension were only associated with advanced fibrosis. Our study demonstrates challenges in identification of early NAFLD given that metabolic syndrome factors were not associated with mild to moderate steatosis.

**Please acknowledge all funding agencies by checking the applicable boxes below:**

None

**Disclosure of Interest:**

None Declared

